# Comment on “Electroacupuncture Improves Intestinal Dysfunction in Septic Patients: A Randomised Controlled Trial”

**DOI:** 10.1155/2021/6487272

**Published:** 2021-02-17

**Authors:** Isabel M. Pacheco-Cerrato, Paula Cobos-Moreno, María A. Castro-Avalos, Juan Fernandez-la-Villa, Juan F. Morán-Cortés, Jose M. Moran

**Affiliations:** Health Science Research Methodology Collaboration Group, Nursing and Occupational Therapy College, University of Extremadura, Cáceres 10003, Spain

With interest, we read a published article in *BioMed Research International* by Meng et al. [1], who investigated the effects of electroacupuncture (EA) at “Zusanli” (ST36) and “Shangjuxu” (ST37) on reducing inflammatory reaction and improving intestinal dysfunction in patients with sepsis-induced intestinal dysfunction with *syndrome of obstruction of the bowels Qi* [1]. The authors stated that EA at ST36-ST37 can reduce inflammatory reaction and has protective effects on intestinal function in patients with sepsis-induced intestinal dysfunction with *syndrome of obstruction of the bowels Qi*. The authors used the Student-Newman-Keuls (SNK) test to compare continuous variables before and after treatment. This test is a relatively powerful stepwise test based on the *q* statistic. The test compares every mean with every other mean in a pair-wise fashion. The SNK test is among a variety of post hoc tests that are used in supplemental examinations after an initial analysis of variance (ANOVA) has been carried out. The test assumes that there are random and independent experiment designs; a breach of this requirement is that the same person is tested in the different groups (dependent design). As the SNK was used by Meng et al. to test changes in different parameters across the follow-up, we consider that there might be inaccuracies in the results presented as the independence assumption between categories was not accomplished in the analysis. Although the test is quite robust to violations of normality or homogeneity of variance, the assumption of independence of observations is critical and should not be infringed. We propose that a more convenient analysis of the data of Meng et al. would be the application of repeated measures ANOVA (equivalent to the one-way ANOVA for dependent measures).
